# Artificial intelligence and the future of maternal and newborn health in low-income countries: advancing equity, early detection, and health system resilience

**DOI:** 10.3389/fpubh.2026.1908521

**Published:** 2026-08-25

**Authors:** Bashir Mohamed Abdi, Shadia Mohamed Ali, Sumeya Ahmed Ali, Mohamed Abdikadir Hussein, Abdulkadir Ibrahim Adow, Sharmake Gaiye Bashir

**Affiliations:** 1 Faculty of Health Sciences and Tropical Medicine, Somali National University, Mogadishu, Somalia; 2 Center for Health Research and Innovation, Somali National University, Mogadishu, Somalia

**Keywords:** artificial intelligence, digital health, early detection, health equity, health system resilience, low-income countries, machine learning, maternal health

## Abstract

Maternal and newborn mortality remain major public health challenges in low-income countries, where poverty, health workforce shortages, limited access to quality care, and weak health systems contribute to preventable deaths and adverse outcomes. Artificial intelligence (AI) has emerged as a promising tool for strengthening maternal and newborn health through improved risk prediction, early diagnosis, clinical decision support, and health system planning. This narrative review examines the potential of AI to advance equity, enhance early detection of complications, and improve health system resilience in resource-constrained settings. Evidence suggests that AI applications can support the identification of high-risk pregnancies, improve the early detection of conditions such as preeclampsia, neonatal sepsis, and respiratory disorders, expand access to obstetric ultrasound services, and optimize resource allocation. AI-enabled digital health platforms also have the potential to strengthen community outreach, referral systems, and quality improvement initiatives across the continuum of maternal and newborn care. However, significant challenges remain, including inadequate digital infrastructure, limited technical capacity, poor data quality, algorithmic bias, and weak regulatory frameworks. The review concludes that AI can contribute meaningfully to reducing maternal and neonatal morbidity and mortality when integrated within broader health system strengthening efforts. Strategic investments in governance, workforce development, digital infrastructure, and equity-focused implementation are essential to ensure that AI technologies support sustainable and inclusive improvements in maternal and newborn health outcomes.

## 1 Introduction

Maternal and newborn health remains a central global public health priority, with preventable deaths and complications concentrated in low and middle-income countries and threatening progress toward Sustainable Development Goals (SDGs). At the same time, rapid digital transformation and emerging artificial intelligence (AI) applications in perinatal care create new opportunities and risks for reshaping how health systems deliver equitable, resilient care across the antenatal, intrapartum, postpartum, and neonatal continuum (1–4).

Marked declines in under-five and neonatal mortality since 2000 coexist with large gaps: neonatal disorders are now the leading cause of death in children under five, and many countries in sub-Saharan Africa and South Asia are off-track for SDG 3.2 targets (2). Effective coverage of reproductive, maternal, neonatal and child health services remains substantially lower in rural, poorer, and lower-HDI populations, underscoring entrenched inequities in access and quality (5) Structural barriers including weak infrastructure, workforce shortages, poor quality of care, and delays in deciding to seek care, reaching facilities, and receiving adequate treatment continue to drive preventable maternal and newborn deaths in developing regions (3, 6, 7).

Against this backdrop, AI-enabled tools are expanding across perinatal care. Applications include pregnancy-risk stratification and decision support for midwives via mobile apps, obstetric point-of-care ultrasound with automated image interpretation, and SMS platforms that support maternal health-seeking behavior and remote monitoring (1, 4, 8–10). Reviews show AI models can enhance early risk detection, diagnosis, and triage for conditions such as preterm birth, preeclampsia, sepsis, and postpartum depression, and can support continuous monitoring through wearables and electronic records (4, 11–13). Evidence from global panel analyses suggests AI adoption is associated with reductions in maternal mortality, particularly in resource-limited settings (14).

However, most AI evidence comes from retrospective, single-center studies with limited representation of low-resource settings, and stakeholders in low and middle-income countries emphasize concerns about algorithmic bias, data security, over-reliance on automation, and fit with fragile health systems (4, 11–13). Achieving Universal Health Coverage (UHC) and SDG maternal and newborn targets will require integrating AI within stronger, better-financed health systems and health insurance arrangements, rather than viewing digital tools as stand-alone fixes (5, 6, 15, 16).

Given this complex, rapidly evolving landscape, a narrative review is well suited to map diverse AI applications across antenatal, intrapartum, postpartum, and neonatal care; situate them within broader inequities, gendered vulnerabilities, and health-system constraints; and synthesize emerging ethical, regulatory, and equity frameworks rather than quantify intervention effectiveness alone (3, 4, 11, 12). The review will first outline the global burden and inequities in maternal and newborn health, then summarize AI use-cases along the perinatal continuum in low-income and other resource-constrained settings, before examining implications for equity, resilience, UHC, and SDGs, and highlighting governance, implementation, and research priorities.

Although this review focuses on maternal and newborn health in low-income countries, evidence from low and middle-income countries and selected high-income settings is included where relevant. This approach was adopted because high-quality studies evaluating artificial intelligence specifically in low-income countries remain limited. Evidence from comparable resource-constrained settings was therefore used to provide a broader understanding of AI applications while interpreting findings within the context of low-income health systems.

## 2 Methods

### 2.1 Study design and reporting approach

This study employed a structured (systematized) narrative review design to synthesize evidence on the application of artificial intelligence (AI) in maternal and newborn healthcare in low-income countries (LICs). Although LICs constituted the primary focus of the review, evidence from low and middle-income countries (LMICs), mixed-country studies, and selected high-income settings was included where findings addressed comparable resource constraints or had clear implications for LIC health systems. This approach was necessary because direct validation and implementation evidence from LICs remains limited. A narrative approach was selected because the evidence encompasses heterogeneous AI technologies, clinical applications, study designs, datasets, validation methods, health-system contexts, and outcome measures and therefore was not suitable for a single homogeneous analytical protocol or meta-analytic synthesis. To reduce the subjectivity and selection bias commonly associated with traditional narrative reviews, the review was conducted using transparent and reproducible procedures, including prespecified search concepts, explicit eligibility criteria, documented study selection, structured data charting, and credibility assessment. Reporting was guided by the principles of the PRISMA 2020 statement, adapted for a narrative synthesis, and by the SANRA (Scale for the Assessment of Narrative Review Articles) criteria. The review was organized around major AI applications in maternal and newborn healthcare, including maternal risk prediction, AI-assisted obstetric ultrasound, clinical decision support, neonatal monitoring and early diagnosis, AI-supported mobile health interventions, and the ethical, equity, governance, and implementation conditions influencing their use in LIC health systems.

### 2.2 Literature search strategy

A systematic literature search was conducted in four electronic databases: PubMed/MEDLINE, Scopus, Web of Science Core Collection, and IEEE Xplore, as shown in Table 1. The search was limited to English-language literature published between 1 January 2021 and 30 June 2026. This period was selected to capture the rapid expansion of contemporary AI applications in maternal and newborn healthcare while maintaining relevance to current technologies, clinical practices, and digital health systems. PubMed/MEDLINE was selected for biomedical, clinical, public health, and health-system literature; Scopus and Web of Science were included to broaden multidisciplinary journal and citation coverage; and IEEE Xplore was searched to capture engineering, computing, signal-processing, medical-imaging, and conference literature that may not be indexed in biomedical databases. Each search strategy combined three concept areas: artificial intelligence and related computational methods; maternal, pregnancy, obstetric, perinatal, neonatal, and newborn healthcare; and low-income, developing, or resource-constrained settings. Controlled vocabulary, Boolean operators, truncation, and database-specific field tags were applied.

**Table 1:** Pathways linking structural determinants, AI-enabled interventions, and potential maternal and newborn health benefits in low-income countries.

Structural determinant or health system barrier	Mechanism increasing maternal or newborn health risk	AI-enabled intervention or digital health strategy	Current evidence base	Potential health system benefit	Potential maternal and newborn health benefit	Policy and implementation priority	Citations
Rural residence, poverty, and geographic inequities	Limited access to antenatal, delivery, and postnatal services	ML models using routine health and DHS-type data to identify high-risk populations	Observational studies and predictive modelling	More targeted resource allocation and outreach planning	May improve service coverage, reduce delays in care, and increase skilled birth attendance	Pro-poor RMNCH policies and rural digital health investment	(5, 7, 80)
Delays in seeking and reaching care (Three Delays)	Late presentation with obstetric complications	AI-supported CHW mobile platforms and referral risk prediction	Early implementation studies	Improved identification and follow-up of high-risk pregnancies	May facilitate earlier referral and timely management of obstetric emergencies	Strengthen CHW digital systems and referral protocols	(31, 81, 82)
Shortage of skilled birth attendants	Limited availability of trained maternity providers	AI clinical decision support, telemedicine, and digital mentoring	Pilot and implementation studies	Enhanced decision support and workforce efficiency	May improve adherence to clinical guidelines and continuity of maternal care	Workforce training and tele-supervision programs	(32, 83)
Poor quality of facility-based care	Inconsistent implementation of evidence-based practices	AI-enabled quality monitoring dashboards	Emerging implementation evidence	Improved monitoring of clinical performance and quality improvement	May contribute to better maternal and neonatal care processes and outcomes	National quality improvement strategies	(80, 81)
Poverty-related social determinants	Increased risk of adverse pregnancy outcomes	AI-assisted integration of clinical and social risk information	Emerging evidence	Better coordination between health and social protection services	May support targeted interventions to reduce adverse birth outcomes among vulnerable populations	Integration of maternal health with social protection policies	(3, 84)
Weak governance and under-resourced health systems	Limited implementation of effective maternal health programs	AI-supported planning, surveillance, and resource prioritization	Mainly conceptual and observational evidence	More efficient planning and health system management	Potential to strengthen health system performance when combined with broader system reforms	Sustainable financing, governance, and AI regulation	(33, 61)

Supplementary sources were identified by examining the reference lists of relevant reviews and key studies and through backward and forward citation tracing. The Consensus academic discovery platform was used only to identify candidate publications and related citations. Its generated summaries were not treated as evidence. Bibliographic information and substantive claims were checked against the original journal record, abstract, or available full text before a source was included.

### 2.3 Eligibility criteria

Studies were selected through an iterative and purposive process guided by their relevance to the review’s objectives and thematic areas. The following criteria informed source selection throughout the review process.

#### 2.3.1 Inclusion criteria

Sources were considered for inclusion if they: were published in peer-reviewed academic journals or were credible policy and technical reports produced by internationally recognized health organizations; addressed maternal health, pregnancy, antenatal or intrapartum care, postpartum care, fetal assessment, perinatal health, neonatal care, or newborn outcomes; evaluated or substantively described an AI, machine-learning, deep-learning, neural-network, computer-vision, predictive-algorithm, or AI-enabled clinical decision-support application; reported empirical findings, validation results, implementation experience, a transparent evidence synthesis, or a clearly documented policy or ethical analysis; were relevant to LICs, LMICs, low-resource settings, or a transferable health problem with explicit implications for LIC health systems; and were available in English within the specified publication period. Outcomes of interest included predictive or diagnostic performance, maternal and newborn morbidity and mortality, early detection, clinical decision support, healthcare access, referral, service utilization, quality of care, workflow effects, implementation feasibility, equity, governance, and sustainability.

Contextual sources addressing maternal and newborn disease burden, social and structural determinants, digital health infrastructure, workforce capacity, data governance, privacy, algorithmic bias, and health-system resilience were included when necessary to interpret whether and how AI could be implemented in LICs. These sources were not treated as direct evidence that AI improved clinical outcomes.

#### 2.3.2 Exclusion criteria

Sources were excluded if they: did not address maternal, fetal, perinatal, neonatal, or newborn healthcare; lacked a substantive AI component; focused only on general digital health without an AI application; described a technical method without a relevant maternal or newborn health application; were editorials, news reports, commercial materials, or opinion pieces without traceable evidence; were duplicate publications; were not available in English; fell outside the specified publication period; or lacked sufficient methodological and bibliographic information for credibility assessment. Protocols and proposals without results were not used as evidence of clinical effectiveness. Evidence from high-income settings was used only when its potential relevance and transferability to resource-constrained health systems were explicitly considered.

### 2.4 Study selection process

Source selection followed an iterative and purposive process suited to structured narrative review methodology. The formal database screening dataset identified 559 records from PubMed/MEDLINE and IEEE Xplore. Five duplicates were removed using DOI and normalized-title matching. During title and abstract screening, 227 records were excluded for failing to meet the eligibility criteria, and one uncertain record was not advanced because insufficient information was available. Therefore, 233 records were removed before full-text assessment, leaving 326 full-text articles to be assessed for eligibility.

During full-text assessment, sources were evaluated according to their direct relevance to maternal and newborn health, substantive use of AI, study design, availability of empirical or validation findings, methodological credibility, evidence maturity, and relevance or transferability to LIC settings. A total of 233 full-text articles were excluded or not retained because they lacked sufficient relevance, did not contain a substantive AI application, provided no empirical or validation findings, lacked methodological transparency, duplicated evidence represented by stronger sources, or had limited applicability to LIC health systems.

**Figure 1:**
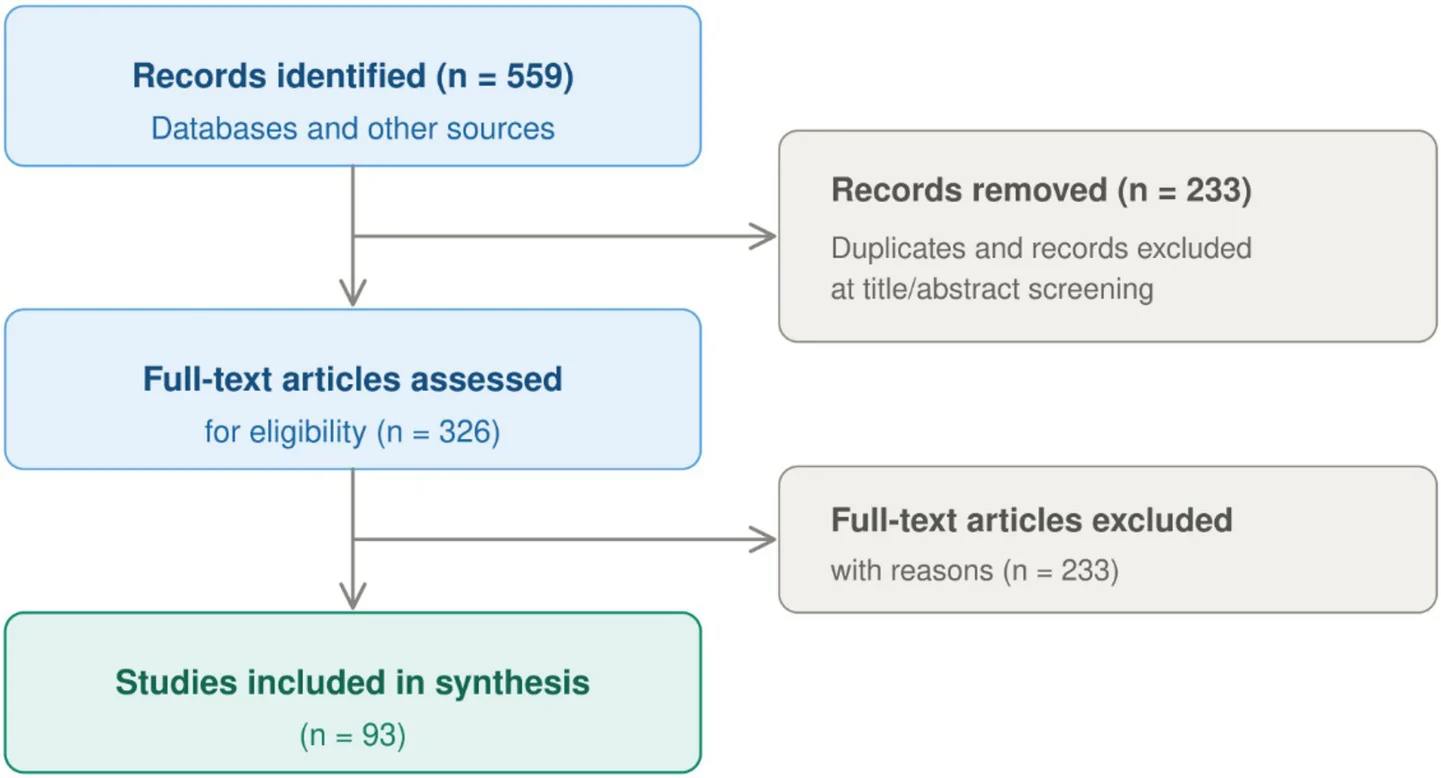
Study-selection process for the structured narrative review. Formal screening totals were based on records exported from PubMed/MEDLINE and IEEE Xplore. Scopus and Web of Science were used to verify multidisciplinary coverage and support citation tracing.

A total of 93 studies and contextual sources were ultimately included in the synthesis, comprising systematic and scoping reviews, AI model-development and validation studies, implementation studies, digital health evaluations, policy analyses, and ethical and governance literature. The study-selection process is summarized in Figure 1.

The Scopus search returned approximately 521 records, while the equivalent Web of Science search was estimated to return approximately 372 records. Because complete record-level exports from these databases were not incorporated into the formal screening matrix, their results were used to verify multidisciplinary coverage, identify candidate publications, and support backward and forward citation tracing. They were not counted as additional unique screened records.

A distinction was maintained between sources used to evaluate AI performance or implementation and sources cited for epidemiological, health-system, ethical, or contextual purposes. Automated and AI-assisted functions were used to organize records, identify possible duplicates, retrieve available metadata, and prioritize records for assessment. These functions did not make final inclusion decisions or replace appraisal of the original publications.

### 2.5 Quality and credibility assessment

Because the review integrated heterogeneous evidence types, including model-development studies, validation studies, implementation research, evidence syntheses, and policy and ethical analyses, a single formal risk-of-bias instrument was not appropriate for the full sample. Instead, each source underwent a structured credibility assessment. The assessment considered: (a) peer-review status or institutional authority of the publishing organization; (b) transparency and appropriateness of the underlying methods; (c) data provenance, sample size, population representativeness, and outcome definition; (d) separation of training and testing datasets and the presence of internal, external, or prospective validation; (e) reporting of model performance, calibration, clinical utility, and implementation outcomes; and (f) relevance and directness of the evidence to LIC or resource-constrained settings.

Reviews were assessed according to the transparency of their search, eligibility, study-selection, appraisal, and synthesis procedures. Greater interpretive weight was given to externally validated empirical studies, transparent evidence syntheses, implementation research, and evidence from recognized academic or international organizations. Studies based on small samples, single-center datasets, retrospective analyses, internal validation alone, or limited methodological reporting were interpreted with appropriate caution. This approach preserved the flexibility of narrative synthesis while avoiding the assumption that all sources provided equally credible or mature evidence.

### 2.6 Data charting

Key information was charted from each included source to support the thematic organization and interpretation of the evidence. For each source, the following details were recorded: publication year; country and income setting; study design; study population and dataset characteristics; maternal or newborn health problem; AI method or technology; intended clinical or health-system function; comparator, where applicable; validation approach; reported predictive, diagnostic, clinical, or implementation outcomes; evidence maturity; major limitations; implementation requirements; equity and governance considerations; and relevance to LIC health systems.

Where available, information was also recorded on discrimination, calibration, sensitivity, specificity, external validation, clinical utility, workflow integration, digital infrastructure, workforce requirements, privacy, algorithmic bias, affordability, and sustainability. The charted information was organized thematically rather than pooled statistically, in keeping with the iterative and interpretive nature of the structured narrative review.

### 2.7 Data synthesis

**Figure 2:**
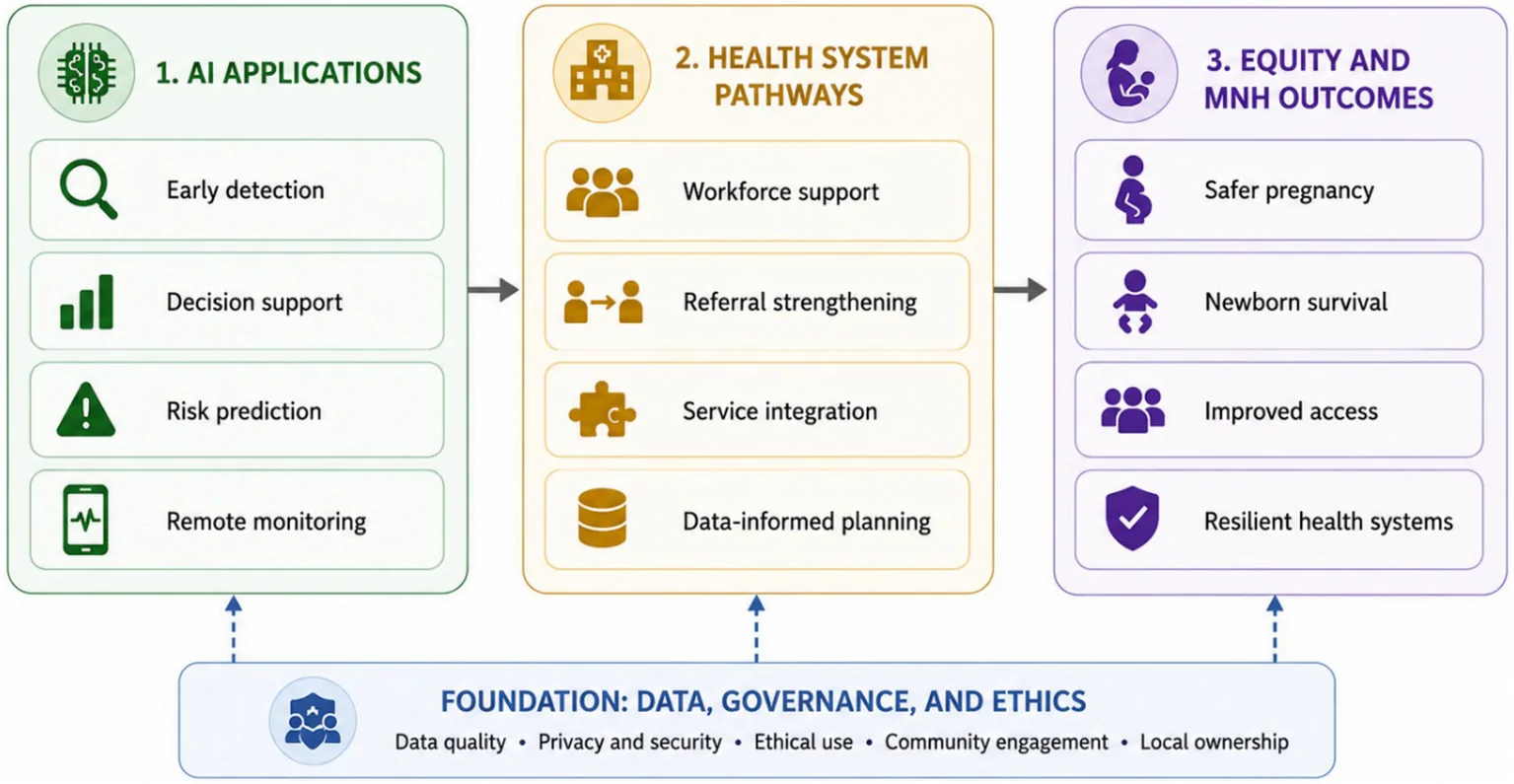
Conceptual framework showing how artificial intelligence applications may influence maternal and newborn health in low-income countries through health-system pathways, supported by foundational requirements of data quality, governance, ethics, community engagement, and local ownership.

A thematic narrative synthesis approach was used to analyze and integrate findings from the included sources. Analysis was guided by the conceptual framework presented in Section 4 (Figure 2), which links maternal and newborn health challenges with major AI applications, health-system enabling conditions, and potential clinical and public health outcomes. The framework provided a structured lens through which evidence was interpreted while accounting for infrastructure, workforce capacity, data quality, governance, affordability, and equity.

Evidence was grouped according to major AI applications and policy themes, including maternal risk prediction, preeclampsia prediction, AI-assisted obstetric ultrasound, maternal clinical decision support, neonatal monitoring and early-warning systems, neonatal sepsis and respiratory-disease detection, and AI-supported mobile health interventions. Cross-cutting themes included evidence maturity, external validation, clinical utility, digital infrastructure, workforce readiness, algorithmic bias, privacy, regulatory access.

Themes were further analyzed in relation to maternal and newborn health outcomes, including early detection, timely referral, clinical decision-making, quality of care, service accessibility, and potential reductions in preventable maternal and neonatal morbidity and mortality. This approach allowed the identification of recurring patterns, promising applications, implementation barriers, policy implications, and gaps in the current evidence base while distinguishing early-stage technological potential from AI interventions supported by mature clinical or implementation evidence.

## 3 Conceptual and analytical framework

This conceptual framework highlights the potential of AI to improve maternal and newborn health in low-income countries. By utilizing clinical, mobile health, and community data, AI applications such as prediction, decision support, and monitoring can support early detection of complications and improve the quality of care. These benefits contribute to stronger health system resilience through better resource allocation, service efficiency, and workforce support. Ultimately, these improvements can lead to more equitable health outcomes, including reduced mortality and improved access to care. However, challenges such as inadequate infrastructure, data privacy concerns, workforce limitations, and funding constraints may affect successful implementation. Addressing these barriers is essential to fully realize the benefits of AI for maternal and newborn health.

## 4 Global burden and epidemiological patterns of maternal and newborn health challenges in low-income countries

Maternal and newborn health continues to carry a heavy epidemiological burden worldwide, with preventable complications during pregnancy, childbirth, and the neonatal period remaining leading causes of death and disability. Global trends show substantial declines in mortality over recent decades but also persistent and stark inequalities by region, socioeconomic status, and setting (12, 14).

Maternal mortality has fallen markedly since 1990, with age-standardized mortality and disability-adjusted life years (DALYs) from maternal disorders decreasing by around 40–60%, yet maternal conditions remain the second leading cause of DALYs among women of reproductive age (17–19). Approximately three-quarters of maternal deaths arise from obstetric hemorrhage, hypertensive disorders, sepsis and other infections, obstructed labor/uterine rupture, and unsafe abortion or miscarriage (17, 18, 20). Hemorrhage is the leading cause of maternal death globally and is particularly dominant in sub-Saharan Africa, while hypertensive disorders contribute a larger share in Latin America, the Caribbean, and some high-income regions (17, 20). Despite progress, low-SDI regions, especially Central and Western sub-Saharan Africa and parts of South Asia, continue to experience the highest maternal mortality ratios and slowest declines (17–19).

For newborns, neonatal disorders now account for nearly half of under-five deaths, with most occurring in the first week of life (21, 22). Global age-standardized incidence, mortality, and DALY rates for neonatal disorders decreased between 1990 and 2021 but remain markedly higher in low-SDI countries (22). Leading causes include preterm birth complications, encephalopathy due to birth asphyxia and trauma, neonatal sepsis and other infections, and complications of low birth weight, which is the dominant risk factor for neonatal infections and overall neonatal disorder burden (22–25). Preterm birth remains the top specific neonatal disorder globally, with declining mortality but heterogeneous trends in incidence and persistently high death rates in Southern and Western sub-Saharan Africa and South Asia (22, 25, 26). Low birth weight and preterm birth contribute substantially to early neonatal deaths and DALYs and are strongly linked to maternal malnutrition, anemia, infections, and inadequate antenatal care in low-resource settings (22–24).

Geographic and population disparities are most pronounced in sub-Saharan Africa, where maternal and neonatal mortality rates are highest and progress is slowest (17, 18, 23). Within countries, rural and remote communities, informal urban settlements, and socioeconomically marginalized groups often have the lowest coverage of antenatal care, skilled birth attendance, and postnatal care (27–29). Conflict-affected and humanitarian settings face amplified risks due to insecurity, displacement, and health service disruption, with elevated maternal and neonatal mortality during and for years after conflict (21). Across these settings, structural contributors include delayed healthcare seeking, limited emergency obstetric and newborn care, weak referral systems, transportation barriers, and severe shortages of skilled health workers, especially for facility-based deliveries and neonatal intensive care (21–24).

Amid these persistent burdens, digital health is rapidly expanding. Mobile health (mHealth) platforms, telemedicine, and broader digital health initiatives have grown over the past 25 years in low and middle-income countries, extending information, remote consultation, and follow-up into underserved areas (28–30). In maternal and newborn health, mobile phone based reminders, decision-support tools, and remote monitoring are increasingly used to support antenatal, delivery, and postnatal care, with evidence of improved antenatal visit attendance and immunization timeliness in some contexts (29, 30). Emerging AI-assisted technologies are beginning to layer onto these platforms for example, risk stratification or decision support but remain at an early stage relative to the scale of maternal and neonatal health needs globally (28, 30).

The findings presented in Table 2 demonstrate that maternal and newborn health outcomes in low-income countries are strongly influenced by structural determinants, including poverty, rural residence, workforce shortages, poor quality of care, and weak health systems. These barriers contribute to delayed care seeking, limited access to skilled services, and increased risks of adverse maternal and neonatal outcomes. AI-enabled interventions offer promising opportunities to address these challenges through risk prediction, targeted resource allocation, decision support, quality improvement monitoring, and integration of social and health data. By strengthening health system efficiency and enabling more precise identification of vulnerable populations, AI technologies can support reductions in preventable maternal and newborn morbidity and mortality. However, the effectiveness of these innovations depends on supportive policies, sustainable financing, workforce capacity building, and equitable digital infrastructure to ensure that technological advances translate into meaningful health gains in underserved settings.

**Table 2:** Current AI applications in maternal and newborn health, evidence base, potential public health benefits, and implementation challenges.

AI application	Target health problem	Current evidence base	Potential public health benefit	Major implementation challenge	Citations
ML prediction models for preeclampsia	Hypertensive disorders of pregnancy	Systematic reviews, retrospective validation studies, predictive modelling	May support earlier identification of high-risk pregnancies and timely intervention	External validation, algorithmic bias, data quality	(85–87)
AI-assisted obstetric ultrasound	Delayed diagnosis of fetal abnormalities and gestational age	Validation studies and implementation research	May expand access to ultrasound services and improve referral decisions	Training, referral capacity, infrastructure	(3, 62, 88)
Deep learning for neonatal monitoring	Preterm infant deterioration	Single-center clinical validation studies	May enable earlier recognition of neonatal deterioration and support clinical monitoring	Monitoring infrastructure and workflow integration	(53, 89, 90)
AI for neonatal sepsis and respiratory disease	Neonatal infections and respiratory distress	Early diagnostic and machine-learning studies	May improve early diagnosis and prioritization of critically ill newborns	Generalizability, deployment in low-resource facilities	(91, 92)
AI-supported mHealth and decision-support systems	Maternal and newborn service delivery	Mixed implementation and observational studies	May improve care-seeking, continuity of care, and health system efficiency	Digital infrastructure, workforce capacity, governance, and equity concerns	(57, 93)

## 5 Structural determinants and health system challenges affecting maternal and newborn health

Structural, social, economic, and health system constraints intersect to shape poor maternal and newborn outcomes in low-income countries. These factors limit timely, quality care across pregnancy, birth, and the postnatal period and disproportionately affect rural, poor, and marginalized women and newborns (31–33).

Poverty and socioeconomic inequality create powerful financial barriers to maternal healthcare. Qualitative syntheses from East Africa show that women struggle to afford antenatal visits, tests, ultrasounds, delivery supplies, and especially transport, with high out-of-pocket costs and lost wages deterring care seeking (34, 35). Financial barriers include direct medical fees, non-medical costs such as food and lodging, indirect income loss, and informal payments, all of which are heavily concentrated among the poorest women (35–37). These costs push women toward home delivery and late or no prenatal care, heightening the risk of preventable complications.

Geographic and infrastructure barriers further constrain access. Women in rural and remote communities face long distances, poor roads, and unreliable or unaffordable transport, making emergency referral particularly difficult (34, 35, 38, 39). Geospatial work in Togo shows that access to EmONC depends heavily on ability to pay for motorized transport; walking-only scenarios leave large populations beyond safe travel times (39). In some settings, fear of the cost of returning a body discourages families from taking critically ill women to facilities (35).

Health workforce constraints are pervasive. Many high-mortality countries fail to reach minimum thresholds of doctors, nurses, and midwives, with severe shortages of obstetric and neonatal specialists and few incentives to work in remote or disadvantaged areas (40). Unequal distribution, high workload, low pay, weak supervision, and poor work environments reduce morale and quality of care, and contribute to disrespect, neglect, and burnout among maternity providers (34, 40–42).

Weak health information and diagnostic systems contribute to delayed or missed diagnosis. Reviews show large gaps in documentation and informational continuity across facilities, with patient-held or home-based records often incompletely or inconsistently filled, undermining safe handover and timely recognition of risk (3, 43, 44). Limited laboratory and imaging capacity, stock-outs of basic equipment, and inadequate referral communication further impede prompt management of maternal and neonatal complications (3, 29, 45, 46).

Gender and sociocultural determinants underlie many of these structural barriers. In East Africa, women’s limited decision-making autonomy, dependence on husbands or relatives for money and permission, and fear of mistreatment deter facility use (34, 35, 46). Harmful norms and stigma, including neglect of female newborns and low perceived value of maternal health, constrain care-seeking and contribute to delayed arrival at facilities (40, 45). Low maternal health literacy and lack of awareness of danger signs compound these effects, particularly among poor, less-educated, and rural women (29, 34, 45).

Digital and technological inequities shape how emerging tools can respond to these gaps. Across LMICs, poor connectivity, electricity shortages, and limited digital literacy restrict the reach and reliability of digital health, including telemedicine and AI-assisted maternal technologies (3, 29, 47, 48). Weak information and communication infrastructure especially affects rural and conflict-affected settings, risking further exclusion of those already facing the highest maternal and newborn risks (29, 47, 48).

## 6 Artificial intelligence applications in maternal and newborn health

Most published evidence on artificial intelligence for maternal and newborn health originates from middle-income and high-income countries, with relatively few implementation studies conducted in low-income countries. Accordingly, this section synthesizes evidence from a range of settings while critically considering its applicability to low-income and other resource-constrained health systems. Where evidence is derived from higher-resource settings, conclusions are interpreted cautiously and discussed in relation to the contextual realities of low-income countries.

AI and other digital innovations are rapidly expanding across the maternal–neonatal continuum, targeting early risk detection, diagnostics, remote care, and workforce support. Most tools remain in development or early deployment, but together they illustrate a shift toward more data-driven, continuous, and personalized care, including in low-resource settings (9, 10, 39, 48).

AI for early risk detection focuses on predicting obstetric and neonatal complications before they become life-threatening. Machine-learning models have been developed to predict preterm birth, preeclampsia, gestational diabetes, postpartum depression, stillbirth, and perinatal mortality using maternal demographics, clinical histories, laboratory data, and prenatal imaging, often achieving higher accuracy than traditional statistical methods (8, 11, 13, 49). In low-resource contexts, AI is being integrated into point-of-care ultrasound to detect fetal growth restriction and placental problems and into SMS systems that support early identification of danger signs and health-seeking behaviors (8). Multi-modal frameworks using Siamese networks and few-shot learning aim to detect fetal anomalies from scarce abnormal imaging data, while ensemble models highlight key maternal and fetal risk factors (50).

AI-assisted diagnostic and clinical decision support systems are emerging across imaging, monitoring, and triage workflows. Deep-learning models now segment fetal anatomy on ultrasound, estimate gestational age and fetal weight, and detect anomalies with high overlap and sensitivity compared to experts, enabling faster, standardized interpretation (12, 13, 50). In neonatology, AI tools support disease prediction, risk stratification, sepsis and retinopathy of prematurity forecasting, and analysis of vital signs, with systems like Hero linked to reduced mortality in very low-birth-weight infants (11, 51–53). Clinical triage algorithms embedded in mobile apps can categorize pregnancies as low, intermediate, or high risk and predict neonatal death with very high reported accuracy, guiding referrals from primary facilities to higher-level care (1).

AI-driven mobile health and remote monitoring are increasingly central to digital transformation. Reviews describe AI-enabled maternal health monitoring using smartphones, wearables, and home-monitoring platforms that track blood pressure, heart rate, glucose, and other parameters and are associated with reductions in maternal mortality and preeclampsia and improved management of gestational diabetes (8, 11, 12, 49). SMS and chatbot systems provide tailored pregnancy education, reminders, and postpartum support, while telemedicine and AI-supported telehealth extend specialist input to remote areas (3, 12, 14, 54).

In health information and surveillance, AI is being applied to large electronic health record and registry datasets to model maternal and neonatal outcomes, predict complications, and support maternal mortality reduction at population level (8, 12, 14, 55). Although explicit maternal mortality surveillance and outbreak-prediction examples are less common, panel data analyses show AI adoption correlating with lower maternal mortality through improved diagnostics, remote monitoring, and automated risk assessment (14). For workforce support, AI-enhanced decision tools and mobile apps assist midwives and frontline workers in risk assessment, diagnosis, and treatment recommendations, while digital training and tele-mentoring platforms aim to address skill gaps and enable task-shifting in settings with few specialists (3, 8, 12, 56).

## 7 Public health and health system implications of AI integration

AI integration into maternal and newborn health systems has implications that extend far beyond individual clinical encounters. At a system and public health level, AI can reshape how risks are detected, resources are allocated, data are used, and inequities are addressed—while also introducing new dependencies and vulnerabilities in low-resource settings (8, 55).

AI-enhanced tools such as Obstetric Point-of-Care Ultrasound and AI-enabled SMS platforms aim to improve early diagnosis and referral, allowing earlier detection of complications and quicker linkage from community to facility care, with potential reductions in maternal complications and improved antenatal and postnatal utilization at population level (8). Observational evidence and global panel analyses suggest AI adoption is associated with declines in maternal mortality, particularly in developing countries, by improving early diagnostics, remote monitoring, and personalized care (14, 57). AI-supported continuity interventions—such as two-way maternal SMS systems and gamified mHealth linked to providers—have increased antenatal visit completion, timely postnatal checks, and reduced neonatal morbidity among disadvantaged groups, indicating system-level strengthening of the continuum of care and referral networks in informal settlements and fragile health systems (58, 59). By providing decision support, triage, and remote supervision for non-specialist providers, AI can enhance system efficiency and extend de facto specialist expertise into underserved areas through telehealth and digital maternal health platforms (54, 59). At the same time, AI-ready digital architectures can improve maternal health surveillance, enabling analysis of large routine datasets for risk prediction and system planning, although LMIC evidence remains sparse and often limited to pilot deployments (49, 60). Equity implications are ambivalent. AI-enabled maternal programs in informal settlements and among refugees show that, when coupled with human providers and targeted to disadvantaged populations, digital tools can improve access and outcomes and may help narrow gaps in service use (58, 59). Yet public health analyses highlight serious risks of widening digital divides, algorithmic bias, and “digital colonialism,” where AI systems trained on data from better-resourced, connected groups systematically underserve rural, poor, and marginalized communities and replicate existing inequities (54). Sustainability concerns are pronounced in low-resource settings, where AI deployments depend on external digital infrastructure, connectivity, continuous model updating, and often subscription-based business models, creating financial and operational vulnerabilities for public maternal health systems (49, 61).

Table 2 highlights the growing role of AI in addressing major maternal and newborn health challenges across low and middle-income countries. AI applications, including machine learning risk prediction models, automated ultrasound interpretation, neonatal monitoring systems, and early diagnostic tools for sepsis and respiratory diseases, have demonstrated significant potential to improve early detection, clinical decision-making, and timely intervention. These technologies can strengthen healthcare delivery by enhancing risk stratification, expanding access to specialized services, and supporting more efficient allocation of limited resources. Nevertheless, their successful implementation remains constrained by challenges such as inadequate digital infrastructure, data quality limitations, algorithmic bias, workforce training needs, and weak regulatory frameworks. Addressing these barriers is essential to ensure that AI innovations contribute equitably and effectively to reducing maternal and neonatal morbidity and mortality, particularly in resource-constrained settings.

## 8 Ethical, implementation, and policy challenges

AI implementation in maternal and newborn care sits within broader healthcare AI ecosystems, so many barriers are shared with other domains but can be especially acute in low-resource, high-vulnerability settings (57, 62).

Data privacy and confidentiality are central concerns because maternal and pregnancy data are highly sensitive, often including reproductive history, fetal information, genetic risk, and disability status. AI’s dependence on large-scale datasets raises “significant ethical concerns, particularly around issues such as data privacy, ownership, and consent” for women, especially those with disabilities, and highlights risks of misuse or future exploitation by third parties (63). Reviews of AI in healthcare more broadly underscore vulnerabilities to unauthorized access, cyberattacks, and unclear data governance, making privacy and security “cornerstone challenges” for trustworthy AI (64, 65).

Algorithmic bias and inequitable performance are major risks because most AI models are trained on non-representative datasets. A scoping review of AI for adverse pregnancy outcomes notes “limited generalizability” from high-income or single-institution cohorts, making algorithms less valid in underserved maternal populations and exacerbating digital inequalities (66). In inclusive maternity care, biased training data can produce “inaccurate or unsuitable recommendations for women with disabilities” and reinforce existing inequities (63). Broader analyses warn that biased medical AI can “exacerbate longstanding healthcare disparities” if not addressed throughout the AI lifecycle (67). and that algorithmic bias in health care requires open, diverse data and systematic bias mitigation (68).

Weak digital governance and regulatory gaps are recurrent barriers. Few LMICs have national AI strategies; governance is fragmented and lags behind technological change, producing “regulatory uncertainty” and a patchwork of standards (64, 69). Obstetric AI reviews call for clear rules on validation, privacy, informed consent, and liability, noting that current legal frameworks are insufficient for the high-stakes context of pregnancy and birth (13, 70). Trustworthy AI requires enforceable, context-specific regulation and clarity on accountability when errors occur, which remains underdeveloped (64, 71).

Operational and infrastructural barriers are especially salient in maternal and newborn care in low-resource settings. Scoping reviews of medical AI deployment and MNCH data science in LMICs highlight unreliable electricity and internet, fragmented or incomplete health data, poor interoperability, and limited local capacity as major constraints on implementation and scale (72). Digital maternal–child health projects in Africa report difficulties translating pilots into policy, overreliance on text-based tools in low-literacy populations, and weak interoperability with routine systems (30). Sustainability is threatened by dependence on donor-funded technologies and short-term projects, with reviews of digital interventions in LMICs describing sustainability as “very complex and multidimensional” and noting barriers related to infrastructure, equipment, and financing for long-term scale-up (73).

Human-level and sociocultural barriers also constrain AI uptake. Digital health implementation reviews find limited digital literacy and resistance to change among health workers as some of the most frequent barriers across technologies, alongside concerns about data privacy and usability (74). In maternal contexts, obtaining meaningful informed consent is particularly challenging in populations with low literacy or social vulnerability, leading to “consent by default” rather than genuine autonomy (66). Women with disabilities may struggle to critically assess AI-generated information, making them more vulnerable to misinformation and undermining informed choice (63). Studies of MNCH digital interventions in peri-urban Pakistan and across LMICs stress the difficulty of building community trust in new systems and ensuring ethical data use where data and digital literacy are low, underscoring sociocultural skepticism as a real barrier (30, 75).

## 9 Future directions and recommendations

Strengthening equitable AI integration into maternal and newborn health systems in low-income countries requires a health-system–centered approach that prioritizes infrastructure, governance, workforce, inclusion, and continuous learning rather than stand-alone technologies. Evidence from maternal health AI, broader LMIC digital health, and global AI governance work provides concrete, context-sensitive directions for policy and implementation (14, 54, 60, 76).

First, governments and partners should invest in foundational digital health infrastructure reliable electricity, internet connectivity, interoperable health information systems, and national digital health platforms that can host multiple tools so maternal AI applications can be deployed safely and at scale (14, 60, 76). Expanding mobile and broadband coverage to rural, informal, and conflict-affected areas, alongside device access and digital literacy support for frontline workers and women, is essential to avoid worsening the digital divide (14, 30, 60, 76). National digital strategies should explicitly link AI for maternal and newborn health with Universal Health Coverage and SDG targets (14, 49, 57).

Second, countries need robust AI governance and ethical regulation tailored to maternal and newborn care. Priority actions include inclusive data practices with diverse, representative datasets; clear rules on privacy, consent, and data sharing; independent audits of algorithms; and transparency, explainability, and accountability mechanisms to address bias and fairness concerns (3, 54, 60, 77). Regulatory frameworks should define validation, monitoring, and re-certification requirements for obstetric AI tools, including performance across marginalized subgroups (13, 78, 79). Equity-focused governance also demands participatory design involving women, especially those with disabilities and from underserved communities, and frontline midwives and nurses so that AI reflects local norms, languages, and accessibility needs (3, 30, 54, 58).

Third, integrating AI into primary-level maternal and newborn services requires strengthening health worker capacity and workflows. Policies should support pre-service and in-service digital and AI training for midwives, nurses, and community health workers; user-centered interfaces; and alignment with existing clinical guidelines to enhance not replace clinical judgment (30, 57, 58, 60). Task-sharing models, AI-assisted telemedicine, and chatbots should be embedded within clear referral pathways, improved transport and communication networks, and functioning emergency obstetric and newborn care (30, 58, 60, 76). National and subnational maternal health surveillance systems, including electronic registries and integrated routine data, should be upgraded to generate high-quality, disaggregated data to power AI, track inequities, and support learning health systems (8, 60, 76).

Fourth, policymakers should foster local innovation, partnerships, and evidence generation. Supporting locally driven AI research, co-development with universities and start-ups, and South–South collaborations can ensure context-appropriate tools and build long-term capacity (54, 57, 58, 76). Public–private partnerships must be structured around public value, open standards, and safeguards against vendor lock-in and unsustainable subscription models, especially for low-resource settings (14, 49, 61, 77). Dedicated funding for implementation research, economic evaluation, and mixed-methods studies of maternal AI tools such as OPOCUS and AI-enabled SMS platforms can inform scale-up decisions and adapt interventions for populations less likely to engage (14, 57, 58, 60). Regionally and globally, collaboration through shared datasets, harmonized standards, and joint capacity-building initiatives can accelerate trustworthy, equity-centered AI adoption while ensuring that low-income countries shape agendas rather than only receive imported solutions (54, 60, 77, 78).

## 10 Conclusion

Artificial intelligence has emerged as a promising tool for addressing persistent maternal and newborn health challenges in low-income countries. By enhancing early risk detection, supporting clinical decision-making, improving disease surveillance, and strengthening health system planning, AI technologies have the potential to accelerate progress toward reducing preventable maternal and neonatal morbidity and mortality. Importantly, AI can help identify underserved populations, optimize resource allocation, and support more equitable delivery of essential maternal and newborn health services.

However, technological innovation alone cannot overcome the structural determinants that drive poor health outcomes. The effectiveness of AI depends on the availability of reliable data systems, adequate digital infrastructure, a skilled health workforce, ethical governance frameworks, and sustained financial investment. Without deliberate efforts to address inequities in access, data representation, and implementation capacity, AI risks reinforcing existing disparities rather than reducing them.

To realize its full public health potential, AI should be integrated within broader maternal and newborn health strategies that prioritize equity, quality of care, and health system strengthening. Future investments should focus on context-specific validation, responsible governance, workforce capacity building, and the development of inclusive digital ecosystems that serve the needs of vulnerable populations. With appropriate safeguards and strategic implementation, AI can become a powerful catalyst for advancing maternal and newborn health, promoting health equity, and building more resilient health systems in low-income countries.
